# High-normal serum carcinoembryonic antigen levels and increased risk of diabetic peripheral neuropathy in type 2 diabetes

**DOI:** 10.1186/s13098-022-00909-7

**Published:** 2022-09-27

**Authors:** Chun-hua Wang, Chao Yu, Lei Zhuang, Feng Xu, Li-hua Zhao, Xiao-hua Wang, Li-yan Ning, Xiu-lin Zhang, Dong-mei Zhang, Xue-qin Wang, Jian-bin Su

**Affiliations:** 1 Department of Endocrinology, Affiliated Hospital 2 of Nantong University, First People’s Hospital of Nantong City, No. 6 Haierxiang North Road, Nantong, 226001 China; 2 Department of Clinical Laboratory, Affiliated Hospital 2 of Nantong University, First People’s Hospital of Nantong City, No. 6 Haierxiang North Road, Nantong, 226001 China; 3 Department of Endocrinology, Second People’s Hospital of Nantong City, No. 43 Xinglong Street, Nantong, 226002 China; 4 Department of Administration, Affiliated Hospital 2 of Nantong University, First People’s Hospital of Nantong City, No. 6, Haierxiang North Road, Nantong, 226001 China; 5 Medical Research Center, Affiliated Hospital 2 of Nantong University, First People’s Hospital of Nantong City, No. 6, Haierxiang North Road, Nantong, 226001 China

**Keywords:** CEA, Risk factor, Neuropathy, DPN, Type 2 diabetes

## Abstract

**Background:**

Increased serum carcinoembryonic antigen (CEA) levels are reported to be associated with various metabolic and inflammatory diseases. This study assessed whether high-normal serum CEA is related to diabetic peripheral neuropathy (DPN) in patients with type 2 diabetes (T2D).

**Methods:**

All subjects received DPN assessment based on neuropathic symptoms, neuropathic signs, and nerve conduction studies to calculate composite* Z* scores of nerve latency, amplitude and conduction velocity (NCV). DPN was confirmed by both at least a presentation of neuropathic symptoms/signs and an abnormal nerve conduction index. Serum CEA levels and other clinical indices were also synchronously detected. Multivariable linear regression analyses were used to determine the independent effects of serum CEA levels on nerve conduction indices, multivariable logistic regression analyses were used to determine the independent impact of CEA levels on the risk of DPN, and receiver operating characteristic (ROC) curve analysis was used to assess the diagnostic capability of CEA levels to discriminate DPN.

**Results:**

We ultimately recruited 402 eligible subjects with normal ranges of serum CEA for this study, and 25.4% (n = 102) were determined to have DPN. After adjusting for other clinical covariates, serum CEA levels were independently associated with the composite* Z* score for latency (*β* = 0.132, *t* = 2.330, *p* = 0.021), amplitude (*β* = − 0.164, *t* = − 2.838, *p* = 0.005) and NCV (*β* = − 0.210, *t* = − 3.662, *p* < 0.001). Moreover, the prevalence of DPN in the first, second, third and fourth quartiles of CEA level was 12.9%, 19.0%, 29.4% and 40.4%, respectively (*p for trend* < 0.001); the corresponding adjusted odds ratios and 95% CIs for DPN in CEA quartiles were 1, 1.47 (0.45–4.82), 1.72 (0.54–5.53) and 4.58 (1.39–15.06), respectively. Furthermore, the optimal cut-off value of high-normal serum CEA to discriminate DPN was ≥ 2.66 ng/mL, with a Youden index of 0.28, sensitivity of 66.67% and specificity of 61.00%.

**Conclusions:**

Increased serum CEA levels within the normal range are closely linked to dysfunction of peripheral nerve conduction and the risk of DPN, and high-normal serum CEA levels are a potential risk factor for DPN in T2D.

**Supplementary Information:**

The online version contains supplementary material available at 10.1186/s13098-022-00909-7.

## Background

Diabetic peripheral neuropathy (DPN), developing in the background of diabetes, is a main precipitating factor for falls, fractures, trauma, foot ulcerations, lower limb amputation and no specific death [1, 2]. Patients with DPN tend to have disability, a poor quality of life, reduced psychosocial well-being and a high burden of health care costs [3, 4]. Although the pathogenesis of DPN is not thoroughly understood, it involves interactions between multiple risk factors in the context of diabetes. Therefore, it is worthwhile to explore additional reliable risk factors associated with DPN pathogenesis, which may help formulate earlier approaches to ameliorate DPN.

Carcinoembryonic antigen (CEA), originally regarded as an oncofoetal antigen, is highly expressed in carcinomas [5]. In contrast, CEA is expressed at low levels in normal tissues of epithelial origin [6]. Moreover, increased levels of serum CEA within the normal or near-normal range are reported to be associated with various metabolic and inflammatory diseases. Increased serum CEA levels are not only cross-sectionally associated with obesity, metabolic syndrome, and leukoaraiosis of the brain [7–9] but can also longitudinally predict cardiovascular events and mortality in the general population [10]. Additionally, serum CEA levels are increased in patients with prediabetes and type 2 diabetes (T2D) and are associated with diabetic complications, such as diabetic nephropathy [11, 12]. Nevertheless, the relationship between serum CEA levels and DPN in T2D has not been well investigated. Therefore, we hypothesize that increased serum CEA levels may be a risk factor for DPN in T2D.

We designed the present study to explore whether increased serum CEA levels within the normal range are associated with an increased risk of DPN in T2D.

## Methods

### Subject recruitment

This study was part of a series we designed to explore potential risks for DPN. We recruited subjects with T2D who visited the Endocrinology Department of First People’s Hospital of Nantong City and Second People’s Hospital of Nantong City between November 2017 and December 2021. The inclusion criteria were as follows: (1) between 20 and 80 years of age; (2) meeting the diagnostic criteria for T2D (2015 Edition, American Diabetes Association) [13]; (3) serum CEA level < 10 ng/mL; and (4) voluntarily agreed to participate in the study. The exclusion criteria were as follows: (1) positive for anti-insulin antibodies; (2) thyroid hormonal abnormality; (3) history of cancer; (4) any possible space-occupying lesions detected by chest X-ray and abdominal ultrasonography; (5) excessive alcohol consumption (excessive alcohol intake, > 40 g daily for women and > 60 g daily for men); (6) severe cardio-cerebral vascular disease (e.g., myocardial infarction); (7) chronic kidney disease and estimated glomerular filtration rate (eGFR) < 60 mL/min/1.73 m^2^; (8) autoimmune disease; (9) acute or chronic infectious disease; (10) connective tissue disease; (11) use of drugs with neurotoxic side effects; (12) deficiency in folate or vitamin B12; (13) neurodegenerative disease; (14) inflammatory demyelinating neuropathy; and (15) spinal or foraminal stenosis. We ultimately recruited 402 eligible subjects with serum CEA levels in the normal range for this study. As the study was initiated and academically supported by First People’s Hospital of Nantong, the study protocol was reviewed and approved by the First People’s Hospital of Nantong. In addition, the processes of the study followed the Declaration of Helsinki, and all participants provided informed consent when recruited.

### Clinical data collection

Collection of clinical data from all participants was carried out by trained clinical staff. Relevant clinical data for statistical analyses included age, sex, body mass index (BMI), systolic/diastolic blood pressure (SBP/DBP), diabetes duration, hypertension status, drinking behavior, statin use, and antidiabetic treatments. Hypertension was identified as reported in our previous study [14]. Drinking behaviour was divided into three categories: no alcohol consumption, alcohol consumption (mild-to-moderate alcohol intake) and excessive alcohol consumption (excessive alcohol intake, > 40 g daily for women and > 60 g daily for men). Antidiabetic treatments were divided into nine categories: drug naive, insulin, secretagogues, metformin, thiozolindiones (TZDs), α-glucosidase inhibitors (AGIs), dipeptidyl peptidase-4 inhibitors (DPP-4Is), sodium-glucose cotransporter-2 inhibitors (SGLT-2Is), and glucagon-like peptide-1 receptor agonists (GLP-1RAs).

Serum was isolated from blood specimens (stored in CAT Serum Clot Activator tubes, Greiner Bio-one) to detect CEA, alanine aminotransferase (ALT), total bilirubin (TBI), albumin, triglycerides (TG), total cholesterol (TC), high-density lipoprotein cholesterol (HDLC), low-density lipoprotein cholesterol (LDLC), uric acid (UA) and C-peptide. Whole-blood specimens were drawn to detect glycosylated haemoglobin (HbA1c). Plasma was isolated to detect glucagon. Serum creatinine was detected to calculate the estimated glomerular filtration rate (eGFR) using the Modification of Diet in Renal Disease equation [15].

Serum CEA levels were measured by chemiluminescence microcarticle immunoassays (CEA Reagent Kit, Architect System, Abbott Ireland Diagnostics Division, Sligo, Ireland) using a fully automated immunoassay analyser (i200SR, Architect, Abbott Laboratories, IL, USA). The precision of the serum CEA assay was tested by determination of intra-assay variation (%CV) and total intralaboratory variation (%CV). Total intralaboratory variation is an overall assessment of intra-assay variation, interassay variation and interday variation. The intra-assay variation in serum CEA was < 2.5%, and the intralaboratory variation was < 2.9%.

### Screening for DPN and nerve conduction analysis

Confirmation of DPN is dependent on both at least a presentation of neuropathic manifestations (neuropathic symptoms or signs) and an abnormal index of peripheral nerve conduction, which is based on the Toronto Consensus Guideline [16]. The screening process for DPN was also described in our previous studies [17, 18].

Neuropathic symptoms and signs were collected by detailed history taking and physical examinations. Neurological symptoms manifested as numbness, imbalance, paresthesia (e.g., cooling sensation) and paroxysmal/persistent pain, such as tingling, burning, electric shock, cutting, and stabbing pain. Neuropathic signs caused by the involvement of large fibre neuropathy were assessed based on reduced or absent ankle reflexes and deep sensation (e.g., vibration perception, pressure perception, balance perception and proprioception) and by involvement of small fibre neuropathy according to superficial sensation (e.g., thermal sensation, haptic sensations and pinprick sensation).

Nerve conduction studies were performed by an experienced neuropathic technician using an electromyogram (MEB-9200K, Nihon Kohden), including the median nerve (MN) and ulnar nerve (UN) of the bilateral upper limbs and the common peroneal nerve (CPN), posterior tibial nerve (PTN), sural nerve (SN) and superficial peroneal nerve (SPN) of the bilateral lower limbs. The nerve conduction parameters included the nerve action potential onset latency, amplitude and conduction velocity (NCV) of the MN, UN, CPN and PTN motor nerves and of the MN, UN, SN and SPN sensory nerves. Data for nerve latency, amplitude and NCV were then *Z* score transformed. Furthermore, a composite* Z* score of latency was generated by taking the average value of the latency *Z* score of all motor and sensory nerves of the upper and lower limbs, as also described in our previous study [18]. In the same way, composite* Z* scores of amplitude and NCV were generated.

### Statistical analysis

The clinical variables were pooled and analysed with SPSS for Windows (Version 25.0, IBM Corp.), and statistical significance was defined as *p* < 0.05.

Clinical variables are shown for all subjects and four subgroups based on the serum CEA quartiles. Normally distributed data were described as the means and standard deviations, skew-distributed data were described as medians and interquartile ranges, and categorical data were described as frequencies and percentages. Serum CEA was skew-distributed and was natural-logarithm transformed (lnCEA). To analyse changes in trends of clinical data among the four subgroups, one-way analysis of variance (ANOVA) with linear polynomial contrasts (*F* value), the Jonckheere–Terpstra test (standard *Z* value) and the chi-squared test with linear-by-linear association (*x*^2^ value) were used, as appropriate.

Pearson’s correlation was employed to assess univariate correlation between serum CEA levels and nerve conduction indices. Moreover, given that HbA1c may exert an effect on these correlations, partial correlation was used to adjust the effect of HbA1c.

Multivariable linear regression analyses were used to determine the independent effects of serum CEA levels on nerve conduction indices, multivariable logistic regression analyses were used to determine odds ratios (ORs) and 95% confidence intervals (CIs) for the risk of DPN in the four subgroups based on CEA quartiles, and receiver operating characteristic (ROC) curve analysis was used to assess the diagnostic capability of CEA levels to discriminate DPN.

## Results

### Clinical features of recruited subjects

The clinical features of all eligible subjects are shown in Table 1. The range of serum CEA levels in all subjects was 0.33–9.72 ng/mL, and those of the first, second, third and fourth quartiles (Q1, Q2, Q3 and Q4) were 0.33–1.73 ng/mL, 1.74–2.54 ng/mL, 2.55–3.70 ng/mL and 3.72–9.72 ng/mL, respectively. Age, SBP and HbA1c increased with ascending serum CEA quartile, whereas the female distribution, albumin and fasting C-peptide decreased. Conversely, diabetes duration, DBP, hypertension history, statin use, ALT, lipid profiles, UA, eGFR and glucagon showed no differences among the four subgroups. Regarding antidiabetic treatments, insulin use tended to increase serum CEA within the normal range (*p* = 0.020), but SGLT-2I use tended to decrease it (*p* = 0.011). The parameters drug naive and use of secretagogues, metformin, TZDs, AGIs, DPP-4Is and GLP-1RAs were comparable among the four subgroups.

**Table 1:** Clinical features of the recruited subjects

Variables	Total	Quartiles of serum CEA levels				*Test statistic*	*p for trend*
		Q1	Q2	Q3	Q4		
Serum CEA (ng/mL) (range)	2.93 ± 1.65 (0.33–9.72)	1.34 ± 0.31 (0.33–1.73)	2.15 ± 0.24 (1.74–2.54)	3.04 ± 0.33 (2.55–3.70)	5.24 ± 1.48 (3.72–9.72)	–	–
lnCEA	0.93 ± 0.54	0.26 ± 0.29	0.76 ± 0.11	1.11 ± 0.11	1.62 ± 0.25	–	–
*n*	402	101	100	102	99	–	–
Age (year)	51.6 ± 9.0	48.3 ± 8.0	51.8 ± 8.2	53.3 ± 9.2	53.1 ± 9.7	16.213^a^	< 0.001
Female, n(%)	161 (40.0)	51 (50.5)	44 (44.0)	36 (35.3)	30 (30.3)	9.995^b^	0.002
BMI (kg/m^2^)	25.0 ± 3.2	25.1 ± 2.9	25.3 ± 3.3	25.1 ± 3.0	24.6 ± 3.6	1.585^a^	0.209
SBP (mmHg)	132.7 ± 16.5	130.0 ± 15.3	131.2 ± 14.4	135.6 ± 17.7	133.9 ± 17.9	4.995^a^	0.026
DBP (mmHg)	79.5 ± 10.5	80.0 ± 10.2	79.2 ± 10.7	79.6 ± 10.6	79.1 ± 10.8	0.223^a^	0.637
Diabetes duration (year)	5.0 (1.0–10.0)	4.0 (1.0–8.0)	5.0 (1.3–10.0)	6.0 (1.0–10.0)	5.0 (1.0–10.0)	1.092^b^	0.275
Antidiabetic treatments							
Drug naive, *n* (%)	43 (10.7)	10 (9.9)	8 (8.0)	15 (14.7)	10 (10.1)	0.288^c^	0.592
Insulin, *n* (%)	170 (42.3)	36 (35.6)	38 (38.0)	46 (45.1)	50 (50.5)	5.455^c^	0.020
Secretagogues, *n* (%)	175 (43.5)	33 (32.7)	47 (47.0)	51 (50.0)	44 (44.4)	3.042^c^	0.081
Metformin, *n* (%)	195 (48.5)	52 (51.5)	48 (48.0)	46 (45.1)	49 (49.5)	0.164^c^	0.686
TZDs, *n* (%)	73 (18.2)	19 (18.8)	20 (20.0)	19 (18.6)	15 (15.2)	0.505^c^	0.477
AGIs, *n* (%)	55 (13.7)	14 (13.9)	15 (15.0)	17 (16.7)	9 (9.1)	0.654^c^	0.419
DPP-4Is, *n* (%)	60 (14.9)	15 (14.9)	17 (17.0)	14 (13.7)	14 (14.1)	0.115^c^	0.735
SGLT-2Is, *n* (%)	17 (4.2)	8 (7.9)	5 (5.0)	3 (2.9)	1 (1.0)	6.413^c^	0.011
GLP-1RAs, *n* (%)	32 (8.0)	7 (6.9)	12 (12.0)	9 (8.8)	4 (4.0)	0.928^c^	0.335
Hypertension, *n* (%)	148 (36.8)	36 (35.6)	30 (30.0)	42 (41.2)	40 (40.4)	1.390^c^	0.238
Alcohol consumption, *n* (%)	99 (24.6)	22 (21.8)	21 (21.0)	26 (25.5)	30 (30.3)	2.414^c^	0.120
Statins uses, *n* (%)	121 (30.1)	25 (24.8)	30 (30.0)	44 (43.1)	22 (22.2)	0.091^c^	0.763
ALT (U/L)	20 (13–28)	18 (12–28)	20 (13–28)	20 (13–30)	18 (13–24)	− 0.239^b^	0.811
TBI (μmol/L)	10.3 (7.6–13.3)	10.8 (7.9–13.7)	9.6 (7.5–11.9)	11.0 (7.7–15.2)	11.0 (7.5–13.2)	− 0.389^b^	0.697
Albumin (g/L)	38.7 ± 3.7	39.6 ± 3.5	38.6 ± 3.1	38.6 ± 4.2	38.1 ± 3.7	7.231^a^	0.007
TG (mmol/L)	1.65 (1.03–2.52)	1.47 (0.97–2.54)	1.72 (1.05–2.38)	1.72 (1.03–2.69)	1.47 (1.05–2.59)	0.180^b^	0.857
TC (mmol/L)	4.36 ± 0.97	4.45 ± 1.01	4.32 ± 0.95	4.41 ± 0.94	4.26 ± 0.97	1.115^a^	0.292
HDLC (mmol/L)	1.23 ± 0.55	1.16 ± 0.30	1.12 ± 0.24	1.16 ± 0.30	1.23 ± 0.55	2.185^a^	0.140
LDLC (mmol/L)	2.71 ± 0.84	2.87 ± 0.92	2.67 ± 0.82	2.63 ± 0.85	2.69 ± 0.80	2.230^a^	0.129
UA (μmol/L)	297 ± 89	289 ± 91	292 ± 88	314 ± 89	293 ± 86	0.682^a^	0.409
eGFR (mL/min/1.73 m^2^)	121 ± 34	124 ± 41	125 ± 35	115 ± 30	118 ± 28	2.234^a^	0.136
Fasting C-peptide (ng/mL)	1.41 (0.85–2.19)	1.78 (1.11–2.19)	1.44 (0.91–2.25)	1.30 (0.81–2.46)	1.05 (0.67–1.75)	− 3.328^b^	0.001
Fasting glucagon (pg/mL)	148.0 (113.3–201.9)	151.1 (115.1–205.8)	152.3 (120.3–210.7)	146.0 (111.8–188.7)	146.8 (132.3–201.1)	− 0.437^b^	0.662
HbA1c (%)	8.09 ± 1.16	7.68 ± 1.01	7.98 ± 1.18	8.29 ± 1.15	8.41 ± 1.21	24.407^a^	< 0.001
Composite *Z*-score of latency	0.03 ± 0.60	− 0.20 ± 0.51	− 0.05 ± 0.56	0.18 ± 0.65	0.20 ± 0.64	29.125^a^	< 0.001
Composite *Z*-score of amplitude	− 0.03 ± 0.66	0.19 ± 0.66	0.002 ± 0.64	− 0.09 ± 0.69	− 0.22 ± 0.66	20.560^a^	< 0.001
Composite *Z*-score of NCV	− 0.03 ± 0.75	0.25 ± 0.63	0.05 ± 0.64	− 0.13 ± 0.77	− 0.32 ± 0.79	17.985^a^	< 0.001
DPN, *n* (%)	102 (25.4)	13 (12.9)	19 (19.0)	30 (29.4)	40 (40.4)	22.767^c^	< 0.001

^a^Linear polynomial contrasts of ANOVA (*F* value), ^b^Jonckheere-Terpstra test (*Z* value) or ^c^linear-by-linear association of chi-squared test (*x*^2^ value) was performed as appropriate

### Correlations between serum CEA and nerve conduction indices

**Fig. 1:**
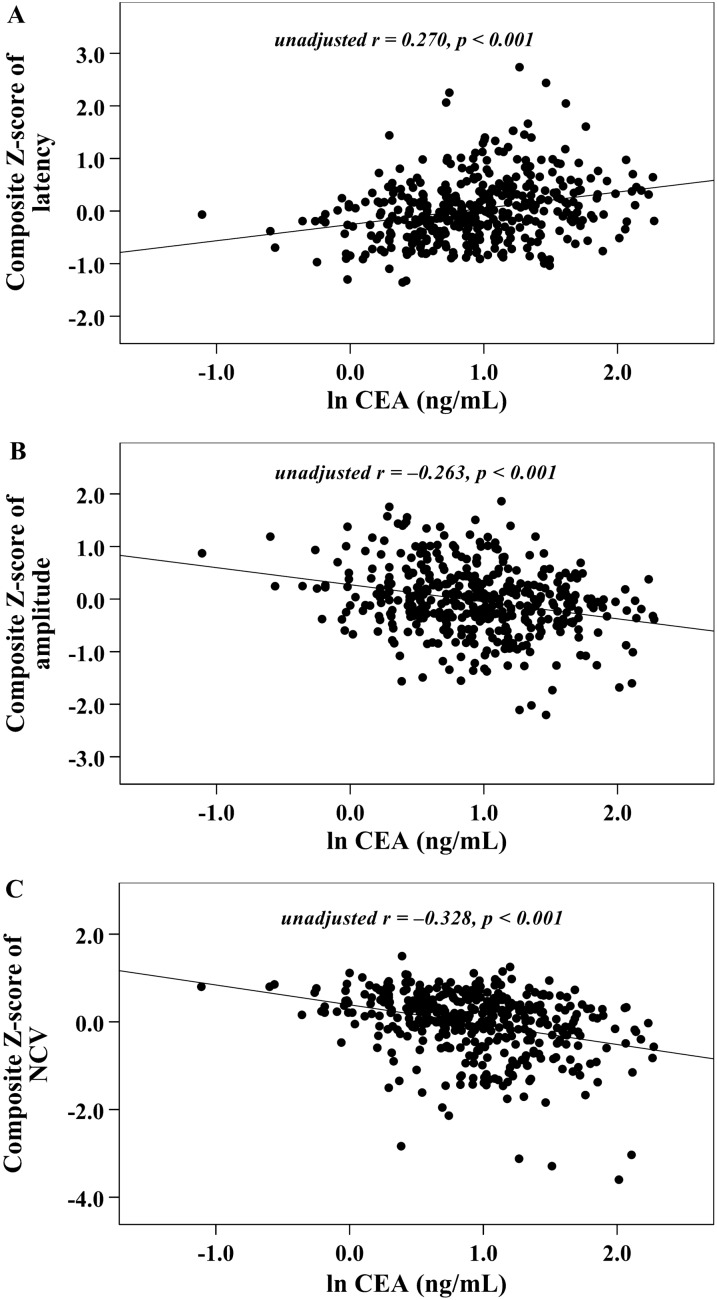
Graphically illustrated correlations between serum CEA levels and nerve conduction indices (**A** composite *Z* score of latency; **B** composite *Z* score of amplitude; **C** composite *Z* score of NCV)

**Fig. 2:**
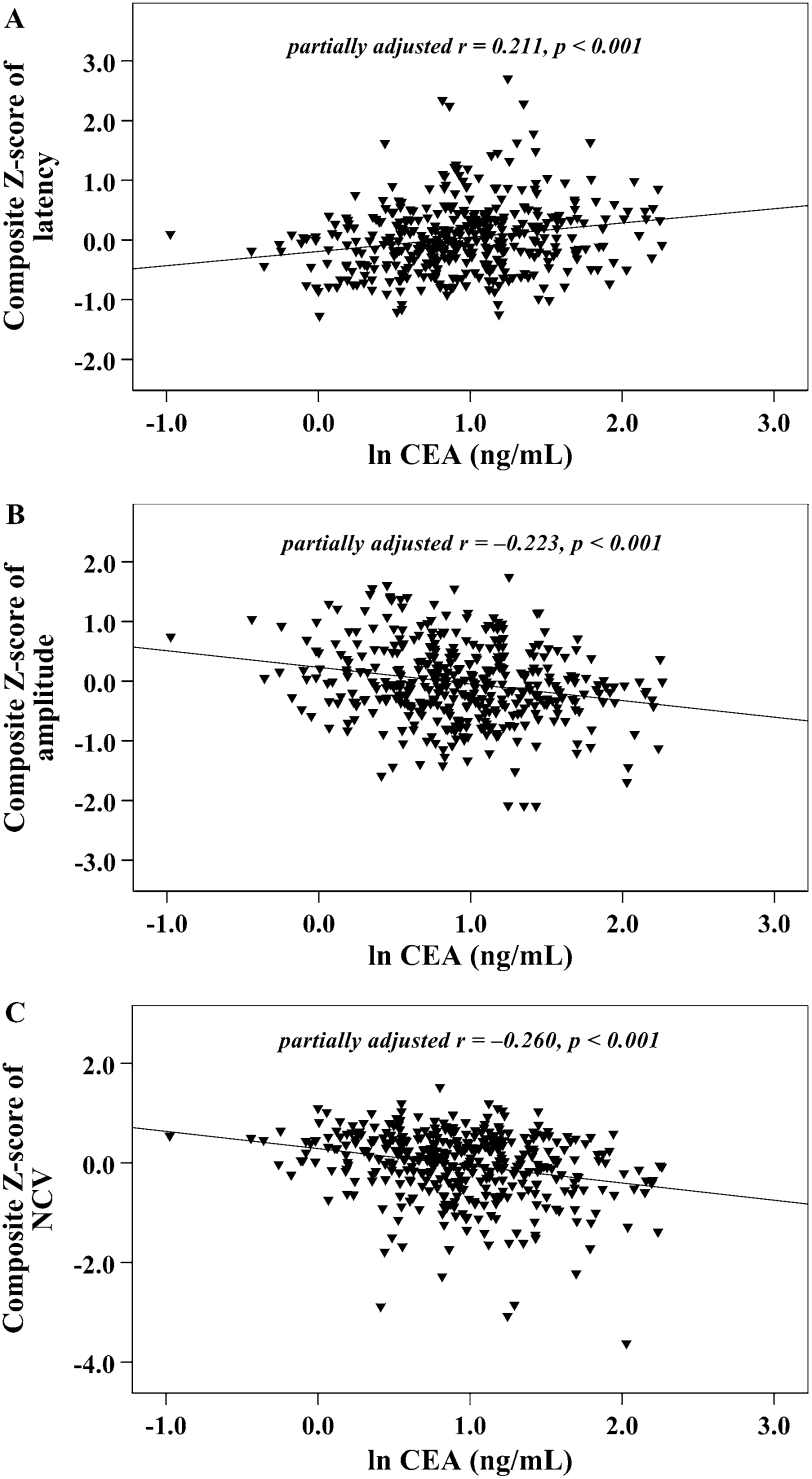
Graphically illustrated correlations between serum CEA levels and nerve conduction indices after adjusting for HbA1c (**A** composite *Z* score of latency; **B** composite *Z* score of amplitude; **C** composite *Z* score of NCV)

With increasing quartiles of serum CEA, the composite *Z* score of latency increased, whereas that of amplitude and NCV decreased markedly (Table 1). Univariate correlation analysis demonstrated serum CEA levels to be linked to the composite *Z* score of nerve latency, amplitude and NCV (*r* = 0.270, − 0.263 and − 0.328, respectively, *p* < 0.001). After controlling for the potential effect of HbA1c on these correlations by partial correlation analysis, serum CEA levels remained linked to the composite *Z* score of nerve latency, amplitude and NCV (*r* = 0.211, − 0.223 and − 0.260, respectively, *p* < 0.001). These correlations are graphically displayed in Figs. 1 and 2.

Moreover, we used multivariable linear regression analyses to determine the effects of serum CEA levels on nerve conduction indices (Table 2). After gradually adjusting for other coagulation function indices and clinical covariates (from model 0 to model 4), serum CEA remained independently associated with nerve conduction indices. According to fully adjusted model 4, serum CEA levels were independently and positively related to the composite* Z* score of latency (*β* = 0.132, *t* = 2.330, *p* = 0.021) and independently and negatively related to the composite* Z* score of amplitude (*β* = − 0.164, *t* = − 2.838, *p* = 0.005) and NCV (*β* = − 0.210, *t* = − 3.662, *p* < 0.001).

**Table 2:** Impacts of serum CEA levels on outcomes of nerve conduction indices by multivariable linear regression analyses

Models	B (95% CI)	*β*	*t*	*p*	Adjusted *R*^2^
Composite Z-score of latency					
Model 0	0.308 (0.200 to 0.417)	0.270	5.608	< 0.001	0.073
Model 1	0.201 (0.097 to 0.305)	0.176	3.803	0.001	0.239
Model 2	0.141 (0.022 to 0.260)	0.119	2.326	0.021	0.342
Model 3	0.147 (0.020 to 0.274)	0.128	2.286	0.023	0.393
Model 4	0.152 (0.023 to 0.280)	0.132	2.330	0.021	0.416
Composite Z-score of amplitude					
Model 0	− 0.324 (− 0.441 to − 0.207)	− 0.263	− 5.461	< 0.001	0.069
Model 1	− 0.197 (− 0.310 to − 0.085)	− 0.160	− 3.462	0.001	0.239
Model 2	− 0.173 (− 0.303 to − 0.042)	− 0.133	− 2.608	0.010	0.341
Model 3	− 0.193 (− 0.338 to − 0.048)	− 0.151	− 2.626	0.009	0.360
Model 4	− 0.209 (− 0.354 to − 0.064)	− 0.164	− 2.838	0.005	0.398
Composite Z-score of NCV					
Model 0	− 0.452 (− 0.581 to − 0.324)	− 0.328	− 6.935	< 0.001	0.107
Model 1	− 0.359 (− 0.487 to − 0.230)	− 0.260	− 5.495	< 0.001	0.204
Model 2	− 0.291 (− 0.441 to − 0.140)	− 0.200	− 3.794	< 0.001	0.299
Model 3	− 0.294 (− 0.449 to − 0.127)	− 0.207	− 3.621	< 0.001	0.369
Model 4	− 0.297 (− 0.453 to − 0.129)	− 0.210	− 3.662	< 0.001	0.406

CEA was natural logarithmically transformed for the regression analyses

Model 0: unadjusted

Model 1: adjusted for age, sex, diabetic duration, BMI, SBP, DBP, hypertension, alcohol consumption, and statins uses

Model 2: additionally adjusted for ALT, TBI, albumin, lipid profiles, UA and eGFR

Model 3: additionally adjusted for HbA1c, fasting C-peptide and glucagon

Model 4: additionally adjusted for antidiabetic treatments

### Risks for DPN of different serum CEA quartiles

After DPN assessment, 25.4% (n = 102) of the recruited subjects were determined to have DPN. The prevalence of DPN in CEA level Q1, Q2, Q3 and Q4 was 12.9%, 19.0%, 29.4% and 40.4%, respectively (*p for trend* < 0.001). Moreover, the ORs and 95% CIs for DPN in CEA level Q1, Q2, Q3 and Q4 were 1, 1.59 (0.74–3.42), 2.82 (1.37–5.80), and 4.59 (2.26–9.31), respectively (Table 3). After adjusting for other clinical covariates by multivariable logistic regression analyses, the corresponding ORs and 95% CIs for DPN in Q1, Q2, Q3 and Q4 were 1, 1.47 (0.45–4.82), 1.72 (0.54–5.53) and 4.58 (1.39–15.06), respectively (Table 3).

**Table 3:** Risk for DPN at different serum CEA quartiles (ORs [95% CIs])

	Q1	Q2	Q3	Q4	*p* value for trend
*n*	101	100	102	99	–
DPN, *n* (%)	13 (12.9)	19 (19.0)	30 (29.4)	40 (40.4)	–
Model 0	1–reference	1.59 (0.74 to 3.42)	2.82 (1.37 to 5.80)	4.59 (2.26 to 9.31)	< 0.001
Model 1	1–reference	1.32 (0.60 to 2.90)	2.22 (1.05 to 4.72)	3.60 (1.72 to 7.56)	< 0.001
Model 2	1–reference	1.42 (0.59 to 3.43)	1.57 (0.65 to 3.83)	3.18 (1.32 to 7.62)	0.010
Model 3	1–reference	1.16 (0.37 to 3.63)	1.56 (0.52 to 4.66)	3.44 (1.12 to 10.55)	0.023
Model 4	1–reference	1.47 (0.45 to 4.82)	1.72 (0.54 to 5.53)	4.58 (1.39 to 15.06)	0.012

Model 0: unadjusted;

Model 1: adjusted for age, sex, diabetic duration, BMI, SBP, DBP, hypertension, alcohol consumption, and statins uses

Model 2: additionally adjusted for ALT, TBI, albumin, lipid profiles, UA and eGFR

Model 3: additionally adjusted for HbA1c, fasting C-peptide and glucagon

Model 4: additionally adjusted for antidiabetic treatments

### Potential capability of serum CEA to discriminate DPN

**Fig. 3:**
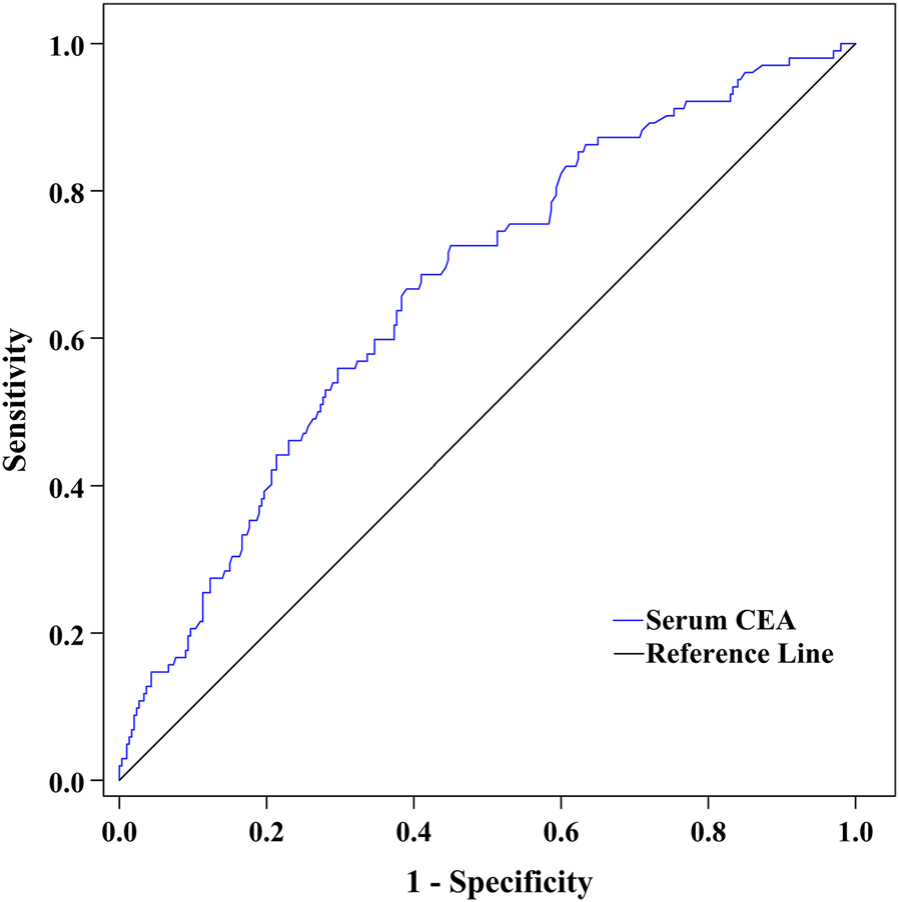
ROC curve exhibiting the capability of serum CEA levels to discriminate DPN (AUC was 0.666 [95% CI 0.617–0.712], optimal cut-off value was ≥ 2.66 ng/mL, Youden index was 0.28, sensitivity was 66.67%, and specificity was 61.00%)

Figure 3 illustrates the ability of serum CEA to discriminate DPN after ROC curve analysis. The area under the ROC curve (AUC) of serum CEA was 0.666 (95% CI 0.617–0.712). Additionally, ROC analysis determined an optimal cut-off value of serum CEA to discriminate DPN of ≥ 2.66 ng/mL, with Youden index = 0.28, sensitivity = 66.67%, and specificity = 61.00%.

### Comparison of serum CEA and HbA1c to discriminate DPN

Because HbA1c was identified as a traditional risk factor for DPN in our previous studies [14, 17], we used ROC analysis to compare the capability of serum CEA and HbA1c to discriminate DPN by the methods of DeLong et al. (Additional file 1: Fig. S1). The AUC of HbA1c in our present study was 0.693 (95% CI 0.646–0.738). After comparing with HbA1c, we found that the capability of serum CEA to discriminate DPN was comparable to that of HbA1c (AUC difference of 0.0277 [95% CI − 0.0516 to 0.107], *Z* = 0.684, *p* = 0.494).

## Discussion

The pathogenesis of DPN is complex and not thoroughly understood [19]. Several risk factors have been proposed for DPN discrimination during the exploration of DPN pathogenesis [20]. We have also initiated series expecting to find new potential risk factors to assist in DPN discrimination. In the series, we routinely tested tumor markers to screen or exclude tumors. To our surprise, we found that increased serum CEA levels within the normal range are related to DPN in T2D. The present study then explored the relationship in depth. The main contributions of the present study are as follows: first, high-normal serum CEA levels were closely connected with the nerve action potential onset latency, amplitude and conduction velocity in patients with T2D; second, the risk of DPN was estimated to be fourfold (OR 4.58, 95% CI 1.39–15.06) higher in patients in the highest quartile of serum CEA than in those in the lowest quartile; third, serum CEA level ≥ 2.66 ng/mL was the optimal cut-off value to discriminate DPN (sensitivity = 66.67%, specificity = 61.00%); fourth, serum CEA and HbA1c, a well-established risk factor for DPN, were comparable in their ability to discriminate DPN.

Increased serum CEA levels have been demonstrated to be associated with a range of unfavourable clinical outcomes under nonmalignant conditions, as well as in malignant tumours, due to its overexpression in adenocarcinomas. In a healthy checkup population (n = 18,131), serum CEA levels correlated positively with age, white blood cell (WBC) count, haemoglobin, aspartate aminotransferase and HbA1c and negatively with BMI and sex (reference: male) [21]. Moreover, increased levels of serum CEA, even within the normal or near-normal range, may be involved in arterial stiffness [22], carotid atherosclerosis [23], abdominal visceral fat accumulation [7], metabolic syndrome [8], metabolic-associated fatty liver disease [24], chronic kidney disease [25], leukoaraiosis [9], and Parkinson’s disease [26] in the general population. In a follow-up study, increased serum CEA may also account for the progression of coronavirus disease 2019 [27], more frequent fracture incidence [28], severity of heart failure (HF), HF adverse prognosis [29], CVD events and all-cause mortality [10]. Furthermore, elevated serum CEA may be an important risk factor for prediabetes and T2D [11], and the levels of serum CEA are closely associated with poor glycaemic control [30] and oxygen desaturation index [31] in patients with T2D. Regarding diabetic complications, higher serum CEA levels may independently contribute to macroalbuminuria in patients with diabetes [12]. In our present study, we found that increased serum CEA is independently associated with increased nerve action potential onset latency and decreased nerve amplitude and NCV in T2D. Patients in the highest serum CEA level quartile showed a fourfold higher DPN risk than those in the lowest quartile. We also calculated that serum CEA ≥ 2.66 ng/mL is the optimal cut-off value to discriminate DPN, with a sensitivity of 66.67% and specificity of 61.00%. Our study adds evidence to support that high-normal serum CEA is a promising risk factor for DPN in patients with T2D.

DPN is a progressive multifactorial diabetic complication. Hyperglycaemia is one of the primary promoters for DPN in T2D, but other risk factors, such as dyslipidaemia, hyperinsulinaemia and inflammation, also contribute [19, 32]. Inflammation is closely connected to hyperglycaemia, insulin resistance and dyslipidaemia in central pathways for DPN [20]. Several cross-sectional and longitudinal studies have recognized the potential clinical relevance of systemic markers of chronic inflammation for DPN. A meta-analysis by Mu et al. [33] showed that a higher level of serum tumour necrosis factor-α (TNF-α) may be independently associated with a higher risk of DPN. Circulating blood leukocyte parameters, such as the neutrophil-to-lymphocyte ratio (NLR) and total WBC count, have been shown to be associated with impaired peripheral nerve functions and increased risk of DPN [34, 35]. Herder et al. [36] revealed in a 6.5-year follow-up study that elevated proinflammatory cytokines, including high-sensitivity C-reactive protein (hs-CRP), interleukin-6 (IL-6), TNF-α, and soluble intercellular adhesion molecule (sICAM-1), can predict the incidence of DPN; hs-CRP, IL-6 and sICAM-1 were also linked to painful DPN independent of metabolic covariates [37, 38]. The serum CEA is actually a pro-thrombotic and pro-inflammatory factor rather than a purely biochemical marker for carcinomas [9]. In our present study, high-normal serum CEA levels were closely associated with DPN independent of glycaemic exposure, dyslipidaemia and other clinical confounders.

Although the role of increased serum CEA in patients with DPN remains unclear, several possible mechanisms may be suggested for the relationship between this marker and DPN pathogenesis. Recent experimental, clinical, and epidemiological studies have pointed towards oxidative stress and inflammatory reactions as important physiopathological mechanisms of diabetic neuropathy [19, 20, 39]. Oxidative stress and inflammatory processes cause DNA damage, endoplasmic reticulum stress, mitochondrial overproduction of superoxide and subsequent apoptosis, and loss of neurotrophic signalling, all of which ultimately lead to nerve dysfunction and neuropathy [20, 40]. Increased serum CEA may serve as an inflammatory mediator and a trigger of oxidative stress in DPN pathogenesis pathways. First, serum CEA levels are closely linked to systemic inflammation markers, such as the total WBC count [41, 42], neutrophil-to-lymphocyte ratio and hs-CRP [26], which reportedly contribute to DPN. Second, some experimental studies have shown that CEA stimulates monocytes and macrophages to release pro-inflammatory cytokines such as IL-1, IL-6, and TNF-α [43, 44]. Third, increased serum CEA is closely associated with oxidative stress markers assessed by serum malondialdehyde (MDA) [45], indicating that increased serum CEA may promote oxidative stress. Additionally, previous studies have revealed that increased serum CEA may have an important role in CNS degeneration, such as leukoaraiosis and Parkinson’s disease [9, 26], which indirectly suggests a role for increased CEA in nerve tissue injury.

The present study should be considered in light of a few limitations. First, due to the cross-sectional design, our data are not sufficient to establish a causal role of increased serum CEA in the development of DPN. In this regard, a longitudinal study is currently being conducted to compensate for this deficiency. Second, the levels of serum CEA correlated with markers of systemic inflammation and oxidative stress, but we did not assess the effect of inflammation and oxidative stress markers on the relationship between serum CEA and DPN. Third, due to the small sample size of patients with painful DPN in our present study, we did not assess the association between CEA levels and the severity of neuropathic pain. Fourth, our results may be affected by the heterogeneity among T2D patients who received multiple antidiabetic agents. We sought to compensate for this limitation by adjusting for antidiabetic agents during the statistical analysis.

## Conclusion

In summary, increased serum CEA levels within the normal range are closely linked to dysfunction of peripheral nerve conduction and the prevalence of DPN, and high-normal serum CEA levels are a potential risk factor for DPN in T2D.

## Supplementary Information

**Additional file 1: Figure S1.** ROC curve comparing the capability of serum CEA levels with that of HbA1c to discriminate DPN.

## Data Availability

Data for this study are available from the principal investigators upon reasonable request.

## Abbreviations

CEA: Carcinoembryonic antigen
lnCEA: Natural logarithm-transformed CEA
Q1: First quartile of CEA levels
Q2: Second quartile of CEA levels
Q3: Third quartile of CEA levels
Q4: Fourth quartile of CEA levels
T2D: Type 2 diabetes
DPN: Diabetic peripheral neuropathy
NCV: Nerve conduction velocity
SBP/DBP: Systolic/diastolic blood pressure
BMI: Body mass index
TZDs: Thiozolindiones
AGIs: α-Glucosidase inhibitors
DPP-4Is: Dipeptidyl peptidase-4 inhibitors
SGLT-2Is: Sodium-glucose cotransporter-2 inhibitors
GLP-1RAs: Glucagon-like peptide-1 receptor agonists
ALT: Alanine aminotransferase
TBI: Total bilirubin
TG: Triglycerides
TC: Total cholesterol
HDLC: High-density lipoprotein cholesterol
LDLC: Low-density lipoprotein cholesterol
UA: Uric acid
eGFR: Estimated glomerular filtration rate
HbA1c: Glycosylated hemoglobin A1c
MN: Median nerve
UN: Ulnar nerve
CPN: Common peroneal nerve
PTN: Posterior tibial nerve
SN: Sural nerve
SPN: Superficial peroneal nerve
