# Supplementary figures and images for: High-normal serum carcinoembryonic antigen levels and increased risk of diabetic peripheral neuropathy in type 2 diabetes
Source: Diabetol Metab Syndr. 2022 Sep 27;14:142. doi: 10.1186/s13098-022-00909-7 (PMC9514694; doi:10.1186/s13098-022-00909-7)

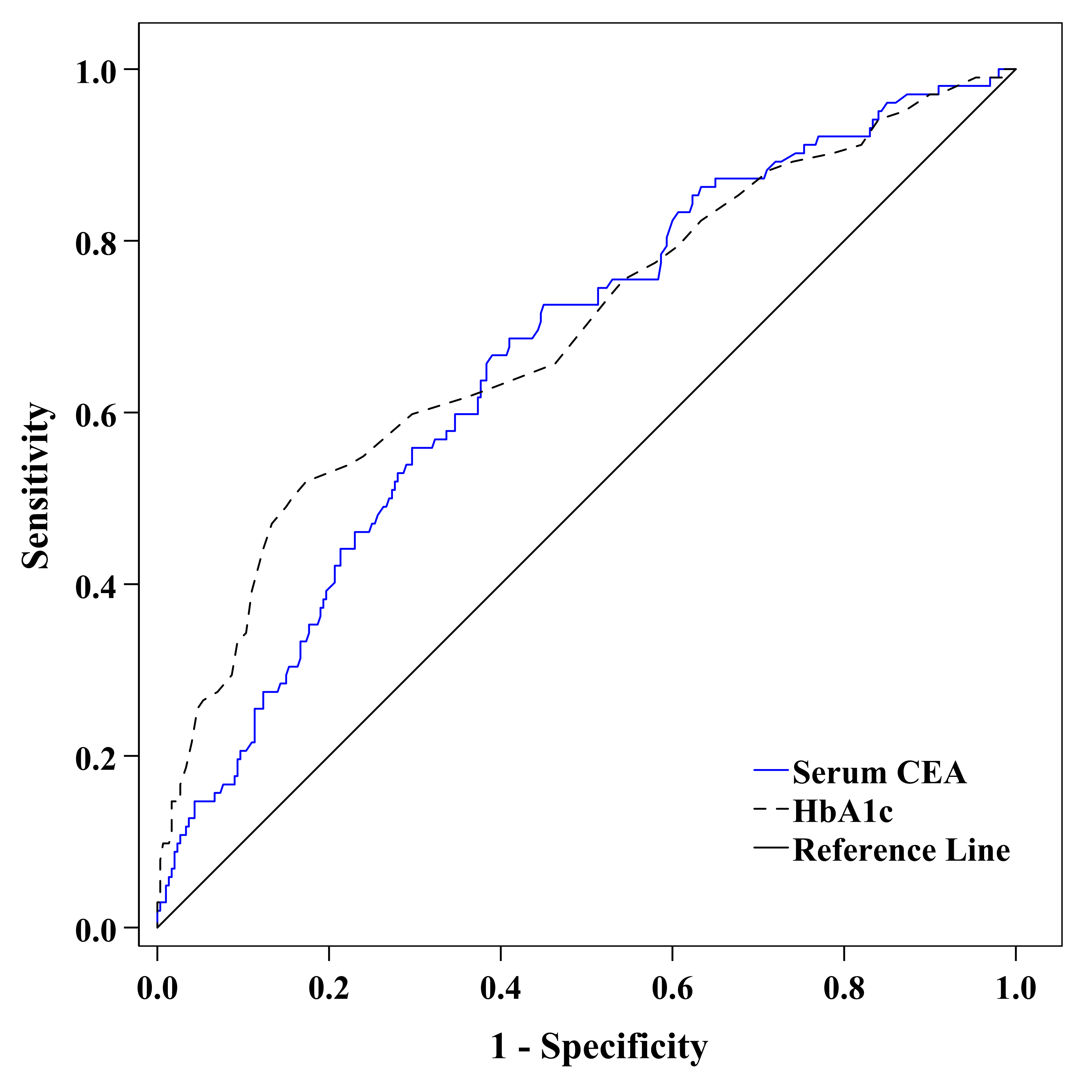

Supplement: Supplementary file 1 — Additional file 1: Figure S1. ROC curve comparing the capability of serum CEA levels with that of HbA1c to discriminate DPN. [file 13098_2022_909_MOESM1_ESM.tif]
